# CROSS-SECTIONAL DIFFERENCES OF PHYSICAL AND PSYCHOSOCIAL MEASURES IN LOW BACK PAIN ACCORDING TO PAIN CHRONIFICATION RISK GROUPS

**DOI:** 10.2340/jrm.v57.42639

**Published:** 2025-08-20

**Authors:** Elisabeth FEHRMANN, Linda FISCHER-GROTE, Julian DIETL, Patrick MAIR, Gerold EBENBICHLER, Thomas KIENBACHER

**Affiliations:** 1 Karl Landsteiner Institute of Outpatient Rehabilitation Research, Vienna, Austria; 2 Department of Pediatrics and Adolescent Medicine, Medical University Vienna, Vienna, Austria; 3 Department of Psychology, Harvard University, Cambridge, MA, USA; 4 Department of Physical Medicine, Rehabilitation and Occupational Medicine, Medical University Vienna, Vienna, Austria

**Keywords:** low back pain, STarT Back screening tool, physical, psychosocial measure, age, gender, BMI

## Abstract

**Objective:**

To investigate whether low-back-pain patients classified based on the risk of pain chronification (low, medium, high) differ in psychosocial and physical function measures, and whether these subgroup differences are moderated by age, gender, and body mass index.

**Methods:**

In this cross-sectional study, 595 Austrian patients with chronic low back pain (68% female; mean age: 53 ± 6.7 years) completed the STarT Back screening tool, visual analogue scale, Roland Morris Disability Questionnaire, Pain Disability Index, 5-level European Quality of Life Questionnaire, Hospital Anxiety and Depression Scale, and Avoidance–Endurance fast screen. Physical function tests assessed maximum trunk strength, trunk range of motion, and hand grip strength, while multivariate analyses of variance evaluated differences among the risk groups.

**Results:**

The intensity of physical and psychosocial problems differed significantly among the pain chronification risk groups. Physical function also varied across subgroups, with the high-risk group exhibiting the weakest muscle strength and the greatest stiffness. Gender significantly moderated the association between pain risk group and trunk strength.

**Conclusion:**

In people of working age with chronic low back pain, the STarT risk of pain chronification was correlated with physical and psychosocial variables. Moreover, this screening tool can be used irrespective of personal factors such as age, gender, and BMI.

## LAY ABSTRACT

This study compared 595 chronic low-back-pain patients among groups at various levels of risk for chronic pain as classified by the STarT Back screening tool across physical and psychosocial measures, and investigated possible interactions with age, gender, and body mass index. The patients completed questionnaires and tests assessing pain, physical and psychosocial variables, and physical performance. Questionnaire results as well as trunk strength, range of motion, and hand grip measures all revealed significant differences among groups at low, medium, and high risk of pain chronicity. These results were partly moderated by gender. The findings suggest that the STarT Back screening tool can predict the likelihood of pain becoming chronic in working-age people independently of gender, age, and body mass index.

Chronic low back pain (cLBP) is the most common chronic health condition worldwide (1), being the primary cause of long-term disability (2). It is associated with extremely high healthcare costs while also reducing an individual’s ability to work or removing them from the workforce entirely (3).

In evidence-based guidelines, the World Health Organization (WHO) recommended a multimodal rehabilitative approach to optimize impaired functioning and health for persons with cLBP. This is supported by a recent concept analysis (4), which aimed to identify the essential attributes of biopsychosocial rehabilitation for cLBP. However, whether all persons with cLBP need rehabilitation remains unclear. Previous research suggested (5) that multimodal rehabilitation should be recommended to cLBP patients at medium or high risk of pain chronification (6, 7), as classified by the STarT Back screening tool (SBST) (6). SBST is a popular, well-regarded, and short patient-reported instrument used to screen for the risk of chronic pain. The risk groups have been shown to correlate with physical and psychosocial variables, such as pain intensity, activity restrictions, depression, fear, and quality of life (QoL) (6, 8, 9), which are also relevant for a classification of LBP patients with the International Classification of Functioning, Disability and Health (ICF) (10). Different care programmes for cLBP stratified by the SBST have been proposed and evaluated, which clearly reduced healthcare costs and sick leave (7, 11). Factors such as age, gender,and body mass index (BMI) should also be considered when planning a rehabilitation programme, as they may markedly affect cLBP patients’ physical functioning and health and rehabilitation outcomes (12, 13–15). However, whether and how such factors affect associations between physical/psychosocial problems and SBST risk classification in working-age populations remains unclear.

In the WHO’s core sets for cLBP, the “muscle function”, “range of motion (ROM)”, and “trunk stability” body functions are important categories to be assessed and addressed in LBP rehabilitation programmes (8). However, there has been limited research relating the risk of chronification of pain as assessed by SBST to relevant ICF-listed physical function categories. Nonetheless, evidence has suggested that LBP patients classified by SBST as being at high risk of pain chronification take longer to bend over and show more protective behaviours compared with the other subgroups (16). In contrast, neither postural stability nor cognitive and proprioceptive functions were affected by the risk of pain chronification (17). Notably, no research has investigated whether SBST subgroups differ in maximum trunk muscle strength, ROM, and hand grip strength. Such assessments provide baseline values for planning physical rehabilitation and are widely covered by health insurance systems.

The primary aim of this study was to analyse whether SBST subgroups of LBP patients differ in physical and psychosocial scores covering important ICF categories. The findings should help characterize LBP patients’ biopsychosocial status prior to intervention and determine which patient subgroups likely most benefit from specific forms and components of rehabilitation. Its secondary aim was to determine whether age, gender, and BMI moderate these group differences, and help to clarify SBST’s utility for assessing pain chronification risk independently of these personal factors.

## MATERIAL AND METHODS

### Participants and study design

Patients referred to an outpatient rehabilitation centre in Vienna, Austria, from various inpatient rehabilitation facilities between April 2021 and February 2023 for progressive strength training-focused rehabilitation were screened for suitability for this cross-sectional study. They used a tablet to complete a self-reported general checklist to assess demographic factors (age, gender, level of education, employment status, marital status), anatomical location, duration and intensity of pain, and functional limitations and comorbidities. Inclusion criteria comprised being ≥ 18 years old and having LBP for ≥ 3 months at a pain intensity of ≥ 30 as rated on a visual analogue scale (VAS 0 to 100) (18). Exclusion criteria were as follows: severe pain (> 60) at anatomical sites other than the lower back; peripheral motor or severe sensory neurological deficits (complete numbness in radicular areas); specific causes of back pain, such as recent spinal fracture, infection or cancer; recent surgery involving the back; osteoporosis; severe mental health issues or an inability to follow verbal instructions in German in a way that would interfere with the study protocol; and pregnancy. Patients underwent a medical examination performed by specialists in physical and rehabilitation medicine and completed the questionnaires on physical and psychological functions within 2 weeks before the physical assessment. Patients with borderline clinical findings could be excluded from the maximum strength testing for medical reasons (e.g., time since specific events causative of LBP, severity of sensory deficits) at the physician’s discretion. Patients were asked not to take analgesic drugs or muscle relaxants within 1 day before functional testing. Shortly before the maximum strength testing, blood pressure was measured. If it was > 160/90 mmHg, second and (if necessary) third measurements were performed after a few minutes of rest. If the result remained above the threshold, the patients were excluded from maximum strength testing. Other exclusion criteria for undergoing maximum strength measurements were intense pain (VAS > 50) on the day of testing or recent night pain, likely indicating significant worsening of the condition.

To determine the minimum sample size, power analysis was conducted (19) covering all outcome variables, which estimated a minimum required sample size of 122 patients to detect medium-sized effects (Cohen’s f^2^: 0.15).

The study complied with the ethical principles of the Declaration of Helsinki and was approved by the Ethics Committee of the City of Vienna (approval No.: 1740/2021). Study conditions were explained comprehensively to eligible participants verbally and in written form, and written informed consent was obtained from all participants.

### Measures

*Physical and psychosocial questionnaires.* LBP chronification risk was appraised with the German version of SBST (10). This questionnaire comprises 9 items, and cut-off values stratify the scores into low, medium, and high chronification risks, which are related to the presence of psychosocial risk factors such as fear, anxiety, catastrophizing or negative future expectations, mood, and bothersomeness of LBP. The questionnaires described below were chosen to represent important body functions according to the ICF LBP core set (8), the generic core sets (20) for sensations of pain (b280), energy and drive functions (b130) and emotional functions (b152), as well as the LBP brief core sets for activities and participation (21).

Pain intensity on the examination day was assessed using VAS ranging from 0 (no pain) to 100 (worst pain imaginable) (covering b280), which has been shown to be a valid and reliable measurement (18).

LBP-related functional disability level was assessed using the validated German version of the Roland Morris Disability Questionnaire (RMDQ) (covering d240, d410, d415, d430, d450, d530, d540, and d640); a maximum of 24 points indicates the most severe perceived back-pain-related health state. The RMDQ has been proven to be valid and reliable (22,23). Perceived back-pain-related disability in 7 domains of life was investigated using the validated German version of the Pain Disability Index (PDI) (covering d640, d760, d845, d850, d859); the maximum score of 70 indicates the most severe perceived disability. The reliability of the PDI has been demonstrated (24).

The 5-Level European Quality of Life Questionnaire (EQ-5D-5L) (25) was used to examine health-related QoL (covering d450, d540, d640, d760, d845, d850, d859, b280, b152). Based on the assessment of problems within 5 life domains, a standardized country-specific index value is calculated, with scores ranging from 0 to 1; higher scores indicate better health-related QoL. The current perceived health status is rated on a numerical scale ranging from 0 to 100, with 100 representing the best imaginable health status. The German version of the Hospital Anxiety and Depression Scale (HADS) (26) was used to identify the presence of anxiety and depression (covering b130 and b152; 27). Back-pain-related coping mechanisms according to the Avoidance–Endurance Model were identified using the valid and reliable Avoidance–Endurance Fast Screen (AEQ-FS) measure (28), which focuses on the persistence of pain and endurance behaviour.

*Physical measures.* The ICF body functions for LBP (b720 and b730) can be classified through the physical measures of maximum back extension and flexion torque and ROM measures (8). A special test device (F110; DAVID Health Solutions, Helsinki, Finland) measured the maximum isometric back extension and flexion strength. A hip fixation mechanism with which this device is equipped allows valid trunk muscle strength measurements. Patients were seated with their knees flexed and their trunks flexed forward at 30° relative to the vertical. The trunk extensor torque was shown on a monitor in real time. Patients were encouraged to undergo a familiarization session and adequate submaximal dynamic warm-up before generating 2 maximum isometric back extensions/flexions under standardized verbal encouragement from expert examiners. A third trial was conducted in cases in which maximum back extensions/flexions varied by > 10%. The better of 2 consecutive attempts was recorded. The F110 device was also applied to assess patients’ trunk ROM in the sagittal plane from the maximum flexed to the maximum extended positions (in degrees). The grip strength of the right and left hands was measured 3 times with a handheld dynamometer. The mean of the best 2 consistent measurements was recorded, and the mean grip strength of the right and left hands was calculated.

### Statistical analysis

All analyses were carried out in R environment (R Foundation for Statistical Computing, Vienna, Austria) (29). SBST subgroups were established using cut-off values. Specifically, those with overall SBST scores of 0–3 were allocated to the low-risk subgroup, while those with ≥ 4 for the psychosocial subscale were allocated to the high-risk subgroup. Those with an overall SBST score > 3 but < 4 on the psychosocial subscale were allocated to the medium-risk subgroup (6). χ^2^ tests were conducted to investigate possible differences in demographic factors among the SBST groups.

To compare levels of physical and psychosocial variables among the SBST groups, multivariate analysis of variance (MANOVA) was performed using VAS and the sum scores of PDI, HADS (anxiety), HADS (depression), EQ-5D-5L, AEQ-FS, and RMDQ as dependent variables and SBST score as an independent variable. Age, gender, and BMI were included in the model as moderators, as shown in the following R syntax: lm(cbind(VAS, pdiScore, hads_fear, hads_depression, eq5d, aeqPpsst, rmdqScore) ~ start_group*Age + start_group*gender + start_group*BMI). The small proportion of missing values within the physical and psychosocial questionnaire data (2.9%) were retained as “NA” in the models. Default case-wise deletion of observations with missing values was applied. Physical function measures were also compared among SBST subgroups using MANOVA with maximum trunk extension and flexion strength, mean hand grip and ROM as dependent variables, SBST score as an independent variable, and age, gender, and BMI as moderators in the following R syntax: lm(cbind(flexion, extension, grip_mean, motion_range) ~ start_group*Age + start_group*gender + start_group*bmi, data = df_physical).

This was followed by MANOVA calls from the “car” package (30). The assumptions of MANOVA (normality, homoscedasticity, homogeneity of variance–covariance) were assessed and showed acceptable results overall. We used Pillai’s trace in all MANOVAs due to its robustness. The MANOVA plots were initially created using the “candisc” package to visualize canonical discriminant analyses (31). For improved readability, the plots were then reconstructed using “ggplot2” (32). Subsequently, based on the individual linear models, effects plots (sjPlot package; 33) were produced, followed by corresponding post hoc tests (emmeans package; 34). To control for inflated Type I errors across 2 MANOVA models, Bonferroni correction was applied to all results by adjusting the significance threshold to α = 0.025 (i.e., 0.05/2).

## RESULTS

### Sample characteristics

A total of 622 patients were eligible for the study and completed the questionnaire assessment. Owing to missing SBST values, 27 patients were excluded from further analyses. Thus, questionnaire data on physical and psychosocial variables from 595 persons with LBP were available. Baseline characteristics of the whole group are presented in Table I. Mean (SD) patient age was 53 (6.66) years, with a range of 26–65 years. Overall, 68.6% of the patients were female. Most participants were employed and had a mean pain intensity of 43.6 (24.58) on VAS on the day they completed the medical examination and questionnaires.

**Table I:** Demographics, pain and disability of chronic low-back-pain patients at baseline

Characteristics	Mean (SD) or *n* (%)
N	595
Age (years)	53.04 (6.66)
Gender (female/male)	408/187 (68.6%/31.4%)
SBST subgroups	
Low risk	380 (63.9%)
Medium risk	140 (23.5%)
High risk	75 (12.6%)
Marital status (%)	
Married	330 (55.5%)
Divorced	120 (20.2%)
Partnered	65 (10.9%)
Single	65 (10.9%)
Educational level* (%)	
Completed compulsory education	64 (10.8%)
Intermediate	412 (69.2%)
University	113 (19%)
Employment status (%)	
Employed	487 (81.9%)
Retired	6 (1%)
Homemaker/maternity leave	2 (0.3%)
Unemployed	84 (14.1%)
Self-employed	6 (1%)
Pain characteristics	
Pain intensity^†^	43.64 (24.58)
Roland Morris Disability Questionnaire	6.68 (5.30)
Pain Disability Index	21.96 (14.18)

* Low/intermediate/high educational level = 9 years/10 to 12 years/> 12 years of schooling;

† Pain scores on a visual analogue scale (0 to 100); SD = standard deviation.

Using SBST, 380 patients were classified as being at low risk of pain chronification, 140 at medium risk, and 75 at high risk. Exploratory χ^2^ tests comparing subgroups across demographic variables revealed significant between-group differences in educational level and employment status. There were fewer individuals who had completed only compulsory education in the low-risk group, and more compulsory school but fewer university graduates in the high-risk group, than expected under independence. Additionally, there were fewer unemployed people than expected in the low-risk group, more in the medium- and high-risk groups, and more people on maternity leave/homemaking as well as fewer employed people in the high-risk group than expected. ANOVA revealed significant differences among the risk groups regarding age, with significantly more young patients in the high-risk group than in the low-risk group.

### Group differences in physical and psychosocial questionnaire data

**Fig. 1:**
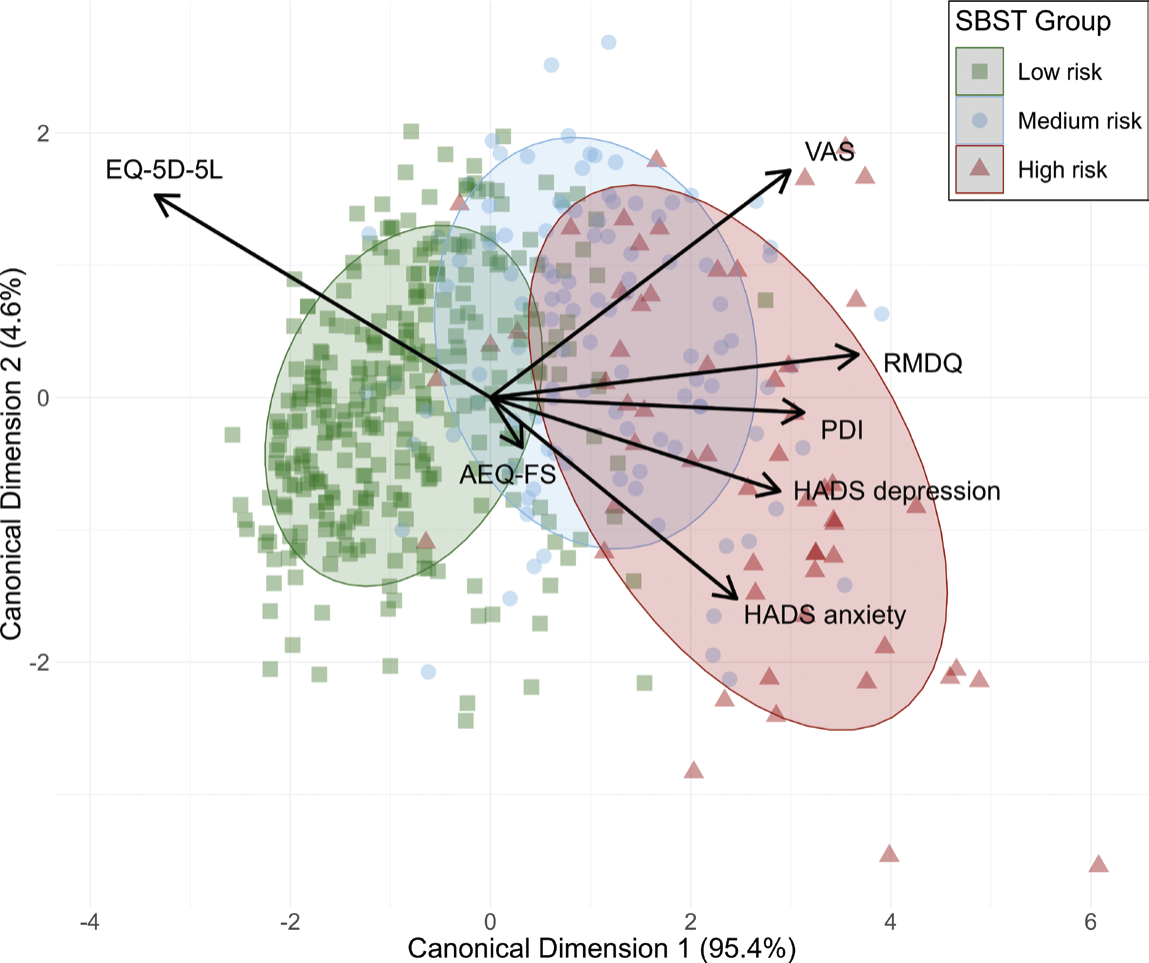
Scatterplot of STarT Back screening tool (SBST) Subgroups for Physical and Psychosocial Variables. Green: low-risk group; blue: medium-risk group; red: high-risk group. SBST: STarT Back screening tool; HADS: Hospital Anxiety and Depression Scale; PDI: Pain Disability Index; AEQ-FS: Avoidance–Endurance Questionnaire Fast Screen; RMDQ: Roland Morris Disability Questionnaire; VAS: visual analogue scale; EQ-5D-5L: European Quality of Life Questionnaire. The ellipses represent the area of 1 standard deviation of the estimated group distributions and visualize the scores for the dependent variables of the 3 SBST groups. The variable vectors are determined by the canonical structure coefficients and represent the correlations of the predictor variables with the canonical variables.

The proportions of missing values were 9% in PDI, 8% in AEQ-FS, 4% in HADS, 2% in RMDQ, 1% in EQ-5D-5L, and 1% in VAS. The correlations among the dependent variables was examined first (see Fig. S1). Fig. 1 presents a scatter plot of the physical and psychosocial scores for each subgroup. The ellipses show the distribution of scores for the dependent variables of the 3 SBST groups.

The MANOVA across all psychosocial variables yielded significant main effects of SBST risk groups for all variables except AEQ-FS but did not identify any significant interactions with age, gender, or BMI (Table II). Pairwise comparisons revealed significant differences among all 3 risk groups for PDI, HADS anxiety, HADS depression, EQ-5D-5L, and RMDQ (Table III). Significant differences between the low- and medium-risk groups, as well as the low- and high-risk groups, were found for VAS.

**Table II:** Results of multivariate analyses of variances of physical and psychosocial variables of the STarT Back screening tool (SBST) subgroups (*N* = 595)

Effect	df	Pillai	F-value	df (num, den)	*p*-value
SBST	2	0.66	31.76	14, 896	< 0.001*
Age	1	0.02	1.49	7, 447	0.171
Gender	1	0.03	2.09	7, 447	0.043
BMI	1	0.11	8.20	7, 447	< 0.001*
SBST:Age	2	0.03	0.91	14, 896	0.553
SBST:Gender	2	0.03	1.13	14, 896	0.325
SBST:BMI	2	0.04	1.25	14, 896	0.236

BMI: body mass index; df: degrees of freedom; Pillai: Pillai’s trace statistic; F-value: multivariate test statistic, representing the ratio of model variance to error variance; num: numerator; den: denominator; *p*-value: significance value, interpreted against a Bonferroni-corrected alpha of 0.0167 (0.05/3) because 3 MANOVAs were performed.

**Table III:** Pairwise comparison of physical and psychosocial variables between STarT Back screening tool (SBST) subgroups

Variables	Contrast	Estimate	SE	df	*t*-ratio	*p*-value
VAS	Low risk vs Medium risk	-27.09	2.24	549	-12.11	< 0.001*
	Low risk vs High risk	-33.79	2.99	549	-11.29	< 0.001*
	Medium risk vs High risk	-6.69	3.36	549	-1.99	0.115
PDI	Low risk vs Medium risk	-13.45	1.29	504	-10.40	< 0.001*
	Low risk vs High risk	-23.00	1.81	504	-12.68	< 0.001*
	Medium risk vs High risk	-9.56	2.01	504	-4.75	< 0.001*
HADS Anxiety	Low risk vs Medium risk	-2.48	0.38	532	-6.47	< 0.001*
	Low risk vs High risk	-6.13	0.52	532	-11.84	< 0.001*
	Medium risk vs High risk	-3.66	0.58	532	-6.31	< 0.001*
HADS Depression	Low risk vs Medium risk	-3.57	0.38	532	-9.33	< 0.001*
	Low risk vs High risk	-6.50	0.52	532	-12.46	< 0.001*
	Medium risk vs High risk	-2.92	0.58	532	-5.01	< 0.001*
EQ-5D-5L	Low risk vs Medium risk	0.18	0.02	549	10.03	< 0.001*
	Low risk vs High risk	0.37	0.02	549	15.02	< 0.001*
	Medium risk vs High risk	0.18	0.03	549	6.66	< 0.001*
RMDQ	Low risk vs Medium risk	0.18	0.02	549	10.03	< 0.001*
	Low risk vs High risk	0.37	0.02	549	15.02	< 0.001*
	Medium risk vs High risk	0.18	0.03	549	6.66	< 0.001*

Contrast: risk group contrast; estimate: differences in adjusted means; SE: standard error; df: degrees of freedom; *t*-ratio: *t*-statistic, the ratio of the difference between group means from the standard error; *p*-value: level of significance, adjusted using Tukey’s HSD and interpreted against a Bonferroni-corrected alpha of 0.0167 (0.05/3) because 3 MANOVAs were performed.

### Group differences in physical measures

Not all patients participated in the physical testing. Overall, 378 out of 595 (64%) patients took part in maximum back strength testing, 565 (95%) in ROM testing, and 511 (86%) in hand grip strength analysis. A total of 33% were excluded from maximum strength testing on the judgement of a physician at a medical examination or due to high blood pressure or high pain levels on the day of maximum performance testing, while 3% of patients provided written informed consent, but either declined to undergo maximum strength testing or were unavailable for the scheduled appointment 2 weeks later. χ^2^ tests revealed that those who did not take part in the physical testing were significantly more frequently in the high- and medium-risk groups than those who did (χ^2^[2] = 56.26, *p* < 0.001). Approximately 74% of patients in the low-risk group participated in the testing, 52% in the medium-risk group, and only 33% in the high-risk group. Upon stratification by gender, 39% of female patients in the high-risk group performed the maximum strength tests, while 20% of male patients did so.

**Fig. 2:**
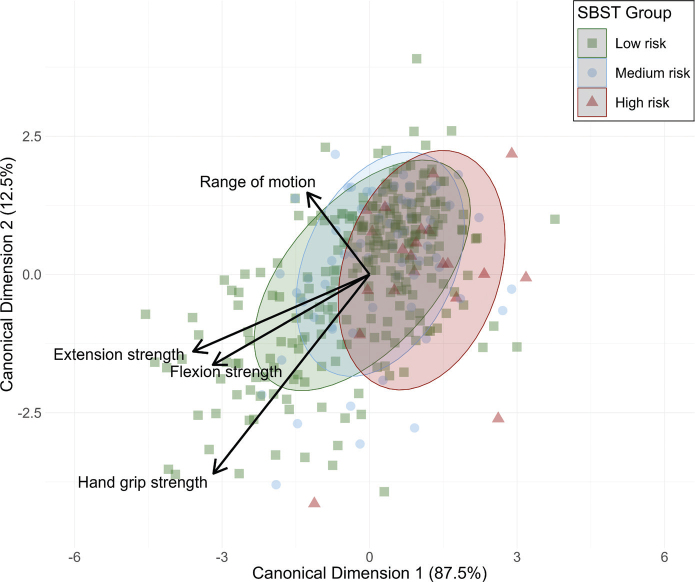
Scatterplot of STarT Back screening tool (SBST) Subgroups for Physical Measures. Green: low-risk group; blue: medium-risk group; red: high-risk group. Range of motion: range of motion of flexion to extension; extension strength: maximum back extension strength; flexion strength: maximum back flexion strength; hand grip strength: mean strength of left and right hand grip measurements. The ellipses represent the area of 1 standard deviation of the estimated group distributions and visualize the scores of the dependent variables for the 3 SBST groups. The variable vectors are determined by the canonical structure coefficients and represent the correlations of the predictor variables with the canonical variables.

The correlation coefficients of the dependent variables reveal moderate correlations among the flexion, extension, and hand grip strength measures (see Fig. S2). The scatter plot in Fig. 2 shows the directions in which the variables influence the ellipses representing the SBST risk groups.

A MANOVA conducted across all variables of the strength and ROM measures yielded statistically significant main effects for the SBST group, as well as significant interactions of SBST group with gender but not with age or BMI (Table IV).

**Table IV:** Results of multivariate analyses of variances of physical measures of the STarT Back screening tool (SBST) subgroups

Effect	df	Pillai	F-value	df (num, den)	*p*-value
SBST	2	0.07	2.90	8, 652	0.003*
Age	1	0.06	4.90	4, 325	< 0.001*
Gender	1	0.71	197.32	4, 325	< 0.001*
BMI	1	0.29	33.14	4, 325	< 0.001*
SBST:Age	2	0.04	1.47	8, 652	0.164
SBST:Gender	2	0.07	2.83	8, 652	0.004*
SBST:BMI	2	0.05	2.0	8, 652	0.044

BMI: body mass index; df: degrees of freedom; Pillai: Pillai’s trace statistic; F-value: multivariate test statistic, representing the ratio of model variance to error variance; num: numerator; den: denominator; *p*-value: significance value, interpreted against a Bonferroni-corrected alpha of 0.0167 (0.05/3) because 3 MANOVAs were performed.

**Fig. 3:**
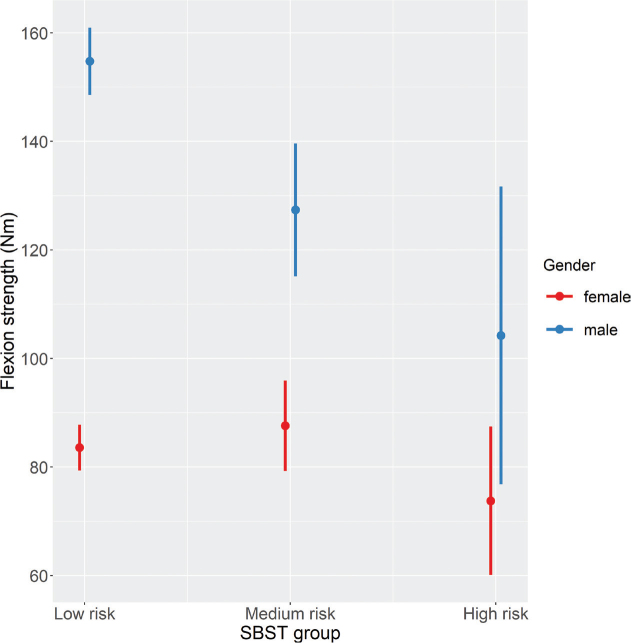
Effects plots for the STarT Back screening tool (SBST) × gender interaction with regard to flexion strength.

For maximum back flexion strength, analyses revealed a significant main effect of SBST risk group (F[2,368] = 4.31, *p* = 0.014). Post hoc tests demonstrated that the low- and high-risk subgroups differed significantly, with LBP patients in the high-risk group showing the lowest maximum back strength scores (Table V). Significant interactions between SBST group and gender were also found (F[2,368] = 9.19, *p* < 0.001), with significant differences in maximum back flexion strength between male and female patients in the low- and medium-risk groups, but not in the high-risk group (Fig. 3, Table SI).

**Table V:** Pairwise comparison of physical measures between STarT Back screening tool (SBST) subgroups

Variables	Contrast	Estimate	SE	df	*t*-ratio	*p*-value
Flexion strength	Low risk vs Medium risk	11.7	4.23	368	2.76	0.017*
	Low risk vs High risk	30.2	7.87	368	3.83	< 0.001*
	Medium risk vs High risk	18.5	8.52	368	2.17	0.078
Extension strength	Low risk vs Medium risk	28.2	9.96	366	2.84	0.013*
	Low risk vs High risk	78.8	18.53	366	4.25	< 0.001*
	Medium risk vs High risk	50.6	20.06	366	2.52	0.032
Hand grip strength	Low risk vs Medium risk	3.45	0.85	498	4.06	< 0.001*
	Low risk vs High risk	4.92	1.12	498	4.38	< 0.001*
	Medium risk vs High risk	1.47	1.27	498	1.16	0.480
Range of motion	Low risk vs Medium risk	1.67	1.20	551	1.40	0.344
	Low risk vs High risk	13.22	1.59	551	8.34	< 0.001*
	Medium risk vs High risk	11.54	1.79	551	6.46	< 0.001*

Contrast: risk group contrast; estimate: differences in adjusted means; SE: standard error; df: degrees of freedom; *t*-ratio: *t*-statistic, the ratio of the difference between group means from the standard error; *p*-value: level of significance, adjusted using Tukey’s HSD and interpreted against a Bonferroni-corrected alpha of 0.0167 (0.05/3) because 3 MANOVAs were performed.

**Fig. 4:**
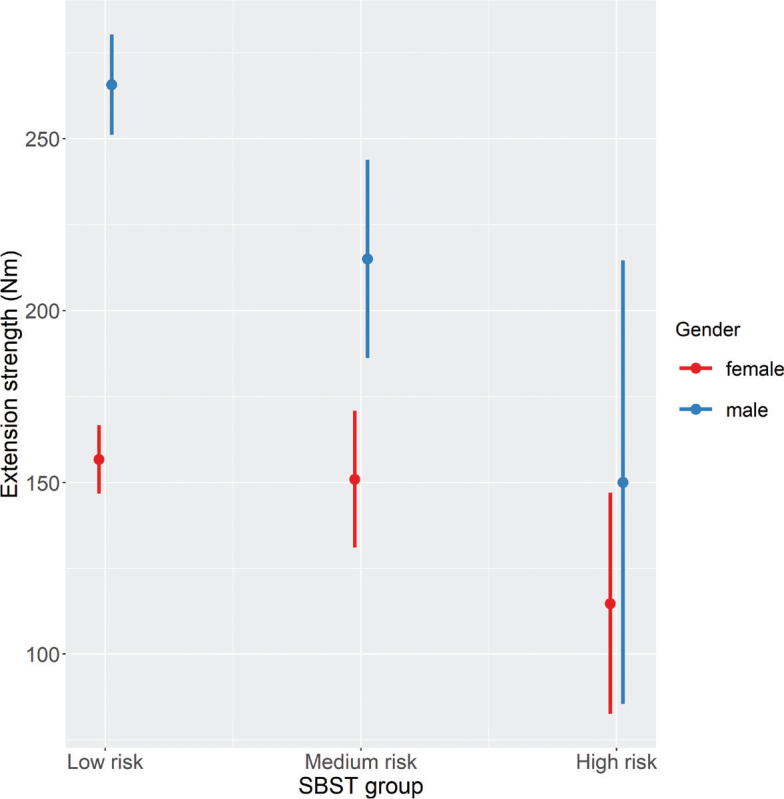
Effects plots for the STarT Back screening tool (SBST) × gender interaction with regard to extension strength.

Considering the maximum back extension strength scores, significant main effects of SBST group (F[2,366] = 9.88, *p* < 0.001) were observed. Subsequent pairwise comparisons showed that the low- and medium-risk groups, as well as the low- and high-risk groups, differed significantly from each other, with the higher risk groups performing significantly worse in maximum strength testing than the other group (Table V). SBST group and gender also interacted significantly (F[2,366] = 3.97, *p* = 0.019), showing significantly lower maximum back extension strength scores for females than for males in the low- and medium-risk groups, but not in the high-risk group (see Fig. 4, Table SII).

For ROM scores, analyses revealed significant main effects of SBST group (F[2,551] = 36.74, *p* < 0.001), with patients in the low- and medium-risk groups reporting significantly greater ROM than those in the high-risk group. Regarding the mean value of hand grip strength, significant main effects could be found according to SBST group (F[2,498] = 14.51, *p* < 0.001), with significantly higher hand grip scores for the low-risk group than for the medium- and high-risk groups.

## DISCUSSION

This study revealed that the SBST risk groups for pain chronification were well correlated with physical and psychosocial measures in people of working age with LBP, while they were not influenced by personal factors (age, gender, and BMI). Furthermore, the SBST risk groups were associated with recommended physical measures (flexion/extension strength, ROM, hand grip strength) that are widely used in cLBP rehabilitation. Significant interactions were observed between gender and SBST risk group when considering maximum trunk flexion and extension muscle strength scores. Notably, a small proportion of patients were available for maximum strength testing in the high-risk group.

Consistent with previous research (6, 9, 10), this study found that SBST-classified risk of pain chronification was strongly associated with the intensity of physical and psychosocial problems. Individuals with cLBP classified as being at high risk for pain chronicity, and to a lesser extent those classified as medium risk, displayed higher pain ratings; more disability, fear, and depression; and a lower QoL than those at low risk. Thus, our findings support previous results (6, 7, 10) indicating that SBST may accurately stratify LBP patients into groups regarding the risk for pain chronification.

Interestingly, in this study, there was no identified relationship between the risk of pain chronification and endurance behaviour as assessed with the AEQ-FS, an aspect that has not been investigated previously. The AEQ-FS examines maladaptive mechanisms for coping with pain, with a major focus on endurance and pain-persistence behaviour (28). Endurance behaviour may not be regarded as a general risk factor for pain chronification, as some studies found a higher risk for pain chronicity in individuals with distress endurance but not in those with eustress endurance behaviour who report feeling positive despite experiencing stress or pain (35).

It is worth noting that BMI was also a major independent risk factor driving pain chronification in cLBP (see Table II), which is corroborated by previous studies (15, 36). However, for clinical use, there is an urgent need to determine whether the SBST risk classification for cLBP can be applied to all cLBP patients or whether it is influenced by particular personal factors. This study’s findings did not reveal any moderating effects of age, gender, or BMI on associations between the risk of pain chronification and the physical and psychosocial variables. This suggests that the SBST risk classification can be applied to working-age cLBP patients without any restrictions.

This study also investigated whether persons with cLBP subclassified into different levels of risk for pain chronification differ in important ICF WHO body function categories, as assessed by measurements of maximum back flexion and extension strength, sagittal ROM (flexion to extension), and hand grip strength. Subjects in the high-risk and partly the medium-risk group exhibited significantly lower trunk muscle strength scores in flexion and extension than those in the low-risk group. This might be explained by the observation that individuals classified as being at high risk for pain chronicity exhibit higher levels of fear-avoidance behaviour, which is known to promote muscle weakening and physical deconditioning via inactivity (37, 38). This is supported by the observation in this study that participants who reported higher anxiety or depression levels were classified more frequently in the high-risk group.

Interestingly, the maximum hand grip strength scores were also found to be significantly lower in the high- and medium-risk groups than in the low-risk group. This finding suggests that fear-avoidance and thus lower motivation to be physically active not only affects trunk muscle strength but may also cause general muscle weakness due to physical inactivity, which also affects hand grip strength, a proxy for a person’s general muscle strength. Another potential explanation relates to physiological associations between emotions and stiffness within the myofascial system (39). Emotional strain, as reported by the high-risk group in particular, could increase tension in the fascia within and around muscles, thereby altering and decreasing the muscle proprioceptive afferent excitatory input to neuromuscular activity overall. Consequently, stress-associated fascial stiffness could catalyse muscle weakness by impairing neuromuscular activity (39, 40). Rehabilitation for such higher-risk patients needs to include psychological interventions to reduce depression and anxiety, influencing deconditioning in a negative way and exercise regimens to optimize functioning and health. Psychological interventions could focus on overcoming maladaptive thoughts and behaviours that affect pain chronification and avoidant behaviour. Furthermore, endurance training could be integrated into the therapy of high-risk patients, which has been proven to achieve antidepressant and anxiolytic effects (41).

This study also revealed that SBST-related differences in maximum back strength scores were affected by the participants’ gender. For the high-risk group, trunk strength scores were similar in males and females, a finding that contrasts with the results from the medium- and low-risk groups, in which males clearly had higher scores than females. This suggests that males at high risk of pain chronification are more likely to be disabled via general muscle deconditioning than females. This is supported by previous research (42) that found a reduction in muscle mass in depressed middle-aged men but not in depressed middle-aged women.

Considering ROM measures, patients at high risk of pain chronification were found to have less flexible spines in terms of ROM compared with the other risk groups. These results appear to be in line with previous findings showing patients in the high-risk group to demonstrate higher pain summation while bending over, longer bending times, and more protective behaviours compared with the low-risk group (16), which is related to greater trunk stiffness and lower ROM. Furthermore, studies found LBP to be associated with back muscle stiffness (43), and an association between stiffness and negative mood (44).

A major limitation of the current study is that there were many more missing data in the high- and medium-risk groups than in the low-risk group for the ICF-related physical function tests. Patients at high risk of pain chronification, who experience and report higher pain and disability levels, psychosocial stressors, and unemployment rates, and who are more likely to have comorbid symptoms, may be less willing to participate in or are more frequently excluded from physical testing procedures, particularly maximum strength testing. However, this may have introduced bias given that participants who did not undergo such testing would presumably have been particularly weak due to deconditioning, comorbidity, or fear of injury. Moreover, the results regarding gender differences in maximum strength measures should be interpreted with caution because the male and female subgroups of the high-risk group were small. However, these results nonetheless align with those obtained in previous research.

Another limitation of our study is that most of the patients were middle-aged and thus of working age. The study results did not show a strong effect of age on the assessments, whereas a wider age range among the subjects may have led to different results. However, LBP poses particular challenges for working-age patients and often results in work duty restrictions, extensive sick leave, or work loss. Therefore, the results of this study, given the age range of the sample, should be valuable.

In conclusion, the results of the study have shown that in people of working age with cLBP, SBST group classification representing the risk of chronification of pain was correlated with physical and psychosocial variables. Thus, this screening tool can be used irrespective of personal factors such as age, gender, and BMI. Maximum muscle strength and ROM were more impaired in the high-risk group than in the lower-risk groups. As the high-risk group of patients showed a greater tendency to withdraw or be excluded from the trunk muscle strength testing, we assert that maximum trunk strength assessments are unnecessary in these patients. These LBP patients likely experience major problems with the deconditioning of trunk muscles and, indeed, of muscles in general. For low-risk patients physical therapy interventions alone might achieve good results, while medium-risk patients probably profit most from a combination of physical and psychosocial therapy. Future longitudinal studies should test the hypothesis that patients at high risk of pain chronification would benefit less from rehabilitation programmes focusing exclusively on physical training therapy and increasing physical strength.

## Supplementary Material




