# Supplementary material for: CROSS-SECTIONAL DIFFERENCES OF PHYSICAL AND PSYCHOSOCIAL MEASURES IN LOW BACK PAIN ACCORDING TO PAIN CHRONIFICATION RISK GROUPS
Source: J Rehabil Med. 2025 Aug 20;57:42639. doi: 10.2340/jrm.v57.42639 (PMC12379722; doi:10.2340/jrm.v57.42639)

Supplementary material has been published as submitted. It has not been copyedited, or typeset by Journal of Rehabilitation Medicine

Fig. S1. Correlation Plot of Dependent Physical and Psychosocial Variables.

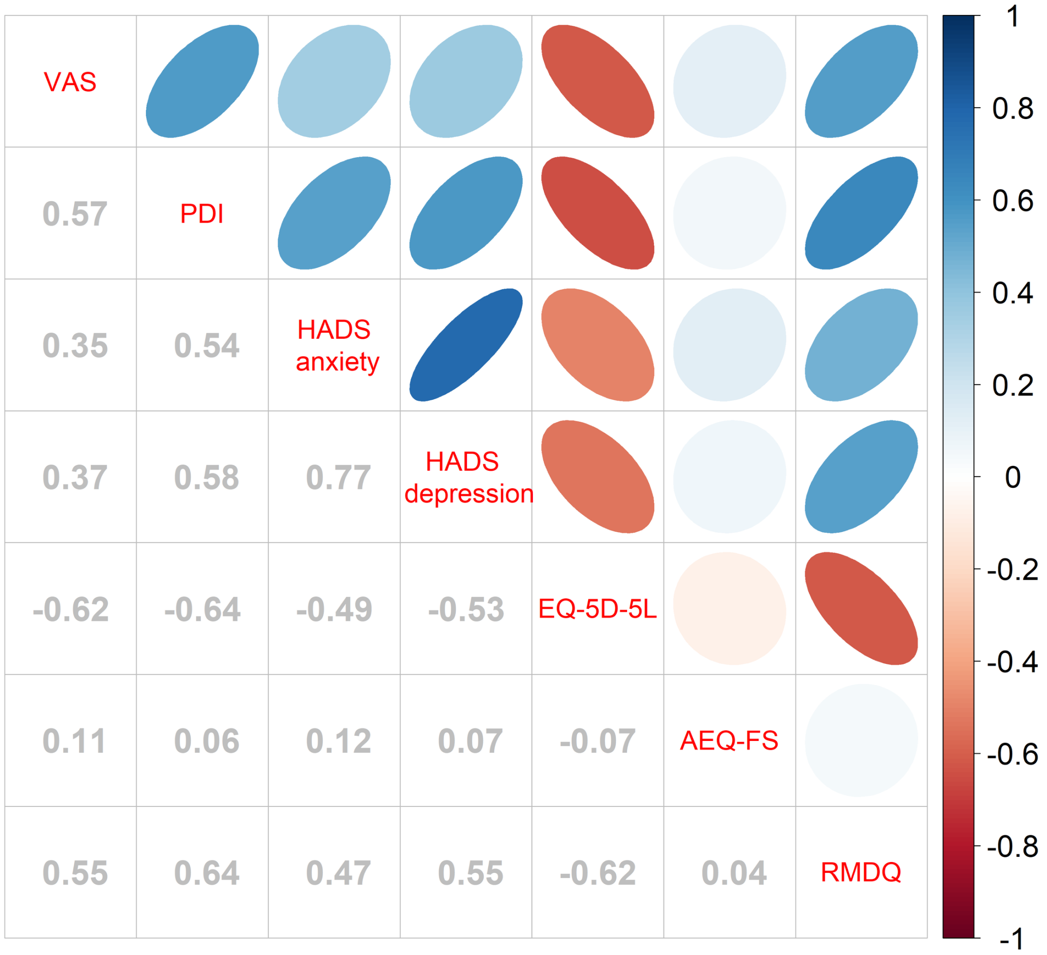

Fig. S2. Correlation Plot of Dependent Physical Measures.

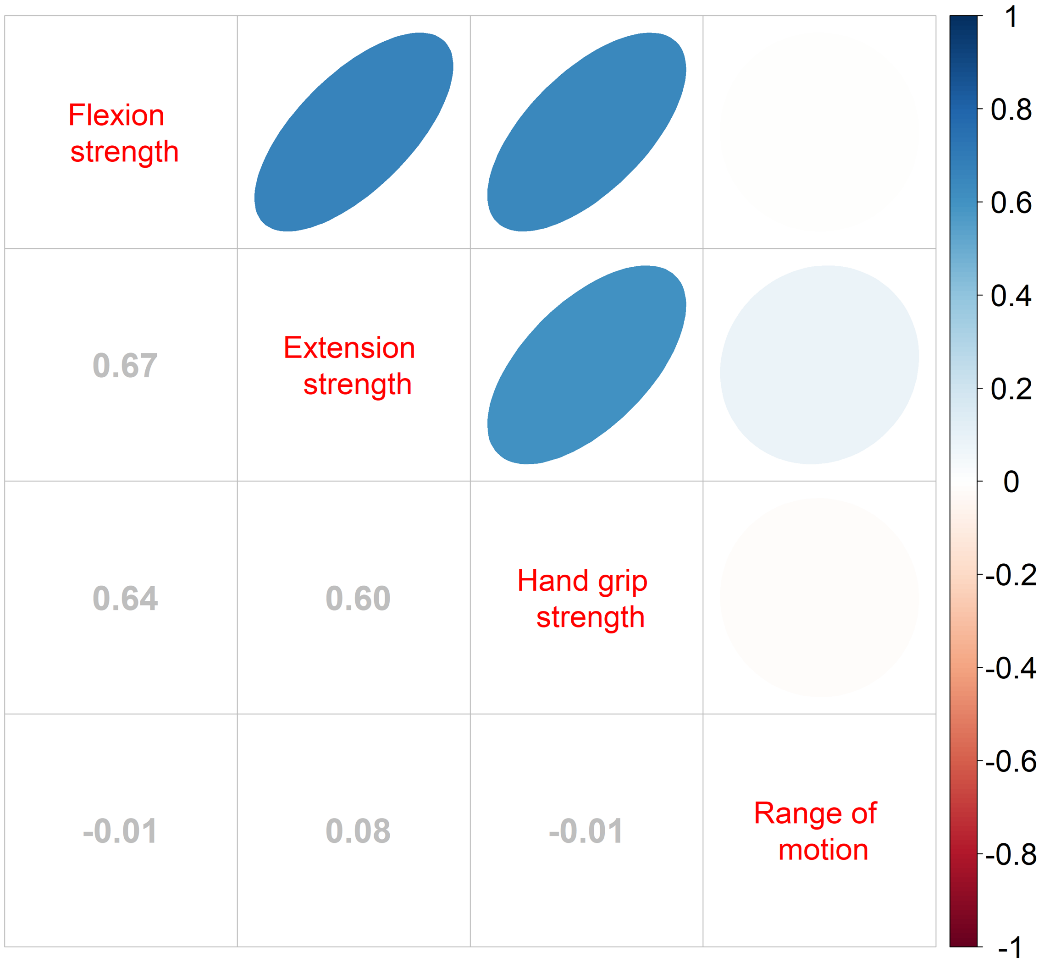

Supplement: Supplementary file 1 [file JRM-57-42639-s1.pdf]
