# Supplementary material for: CROSS-SECTIONAL DIFFERENCES OF PHYSICAL AND PSYCHOSOCIAL MEASURES IN LOW BACK PAIN ACCORDING TO PAIN CHRONIFICATION RISK GROUPS
Source: J Rehabil Med. 2025 Aug 20;57:42639. doi: 10.2340/jrm.v57.42639 (PMC12379722; doi:10.2340/jrm.v57.42639)
Supplement: Supplementary file 2 [file JRM-57-42639-s2.pdf]

Supplementary material has been published as submitted. It has not been copyedited, or typeset by Journal of Rehabilitation Medicine

Table SI: Pairwise Comparisons of Gender by SBST Group with Respect to Flexion Strength

SBST Group	Contrast	Estimate	SE	df	t-ratio	p-value
**Low-risk group**	Female vs. male	−71.2	3.82	368	−18.63	< .001 *
**Medium-risk group**	Female vs. male	−39.8	7.50	368	−5.30	< .001 *
**High-risk group**	Female vs. male	−30.4	15.89	368	−1.92	0.056

Notes: SBST = STarT Back screening tool; estimate = differences in adjusted means; SE = standard error; df = degrees of freedom; t-ratio = t-statistic, the ratio of the difference between group means to the standard error; p-value = level of significance, adjusted using Tukey's HSD and interpreted against a Bonferroni-corrected alpha of 0.0167 (0.05/3) because three MANOVAs were performed.

Table SII: Pairwise Comparisons of Gender by SBST Group with Respect to Extension Strength

SBST Group	Contrast	Estimate	SE	df	t-ratio	p-value
**Low-risk group**	Female vs. male	−109.0	9.0	366	−12.11	< .001*
**Medium-risk group**	Female vs. male	−64.0	17.8	366	−3.60	< .001*
**High-risk group**	Female vs. male	−35.2	37.4	366	−0.94	0.347

Notes: SBST = STarT Back screening tool; estimate = differences in adjusted means; SE = standard error; df = degrees of freedom; t-ratio = t-statistic, the ratio of the difference between group means to the standard error; p-value = level of significance, adjusted using Tukey's HSD and interpreted against a Bonferroni-corrected alpha of 0.0167 because three MANOVAs were performed.
